# Gender, Work, and Health for Trans Health Providers: A Focus on Transmen

**DOI:** 10.5402/2012/161097

**Published:** 2012-12-17

**Authors:** Judith A. MacDonnell, Alisa Grigorovich

**Affiliations:** ^1^School of Nursing, York University, Room 322, HNES Building, 4700 Keele Street, Toronto, ON, Canada M3J 1P3; ^2^School of Women's Studies, York University, 206 Founders College, 4700 Keele Street, Toronto, ON, Canada M3J 1P3

## Abstract

Well-documented health research points to trans people's vulnerability to health inequities that are linked to deeply embedded structural and social determinants of health. Gender and work, as social determinants of health for trans people, both shape and are shaped by multiple factors such as support networks, social environments, income and social status, shelter, and personal health practices. There is a gap in the nursing literature in regards to research on work and health for diverse trans people and a virtual silence on the particular issues of trans-identified health providers. This qualitative study used comparative life history methodology and purposeful sampling to examine links among work, career, and health for transmen who are health providers. Semistructured interviews were completed with four Canadian transmen involved in health care professional and/or practice contexts with diverse professions, age, work, and transitioning experiences. Critical gender analysis showed that unique and gender-related critical events and influences shape continuities and discontinuities in their careerlives. This strength-based approach foregrounds how resilience and growth emerged through participants' articulation with everyday gender dynamics. These findings have implications for nursing research, education, and practice that include an understanding of how trans providers “do transgender work” and supporting them in that process.

## 1. Introduction

Despite greater public and professional awareness of sexual and gender diversity and more positive work climates in recent years, lesbian, gay, bisexual, transgender, and queer (LGBTQ) people continue to encounter invisibility, overt and more subtle discrimination and violence, and other barriers to relevant, respectful care. According to the Registered Nurses Association of Ontario (RNAO), “Sexual diversity relates to sexual orientation (lesbian, gay, bisexual, and heterosexual) and gender diversity refers to gender identity (e.g., transgender) which refers to one's sense of being male and/or female or neither” [1]. This last decade has brought greater attention to transgender health, the “T” in LGBTQ, in health care and research has shown that ethnicity, race, geography, age, education, and other social categories across genders influence how particular trans-identified individuals experience and understand their health. Typically, transgender is used in the broadest sense to refer to people who transgress gender norms in some way [2]. In this paper, “…[*t*]*rans* refers to transgender, transsexual, gender nonconforming, and gender questioning (people)” [3] and transmen to those who identify with a masculine trans identity. Within trans communities, individuals express their gender and subscribe to identities in a variety of ways. These include transmen, transwomen, and genderqueer, and trans identities may shift over a lifetime [4]. Gender expression and/or transition processes can involve dress, electrolysis, and/or sex reassignment surgery.

Encounters with violence, transphobia, and discrimination related to sexuality and gender norms across home, community, work, and educational settings, and challenges to finding meaningful social support, shape trans people's disclosure with respect to trans issues and identities, their health status, and access to care [5–7]. Considerable hurdles also exist to locating sensitive and knowledgeable health providers, trans-positive services and obtaining hormone therapy or surgery if so desired, especially outside of large urban cities [6, 8]. All of these factors contribute to trans people's vulnerability to health inequities.

As social determinants of health, employment, and working conditions for trans people both shape and are shaped by multiple factors such as support networks, social environments, income and social status, shelter, and personal health practices [6, 7, 9, 10]. Decisions to transition in the workplace are informed by many factors that include underemployment, available support systems, and weighing the costs of job-loss or unpredictable discrimination that mark many environments for trans people [8, 9, 11]. Certainly income level and access to education are also relevant to trans individuals' capacity to enter the professions.

LGBTQ workplace issues have recently become high profile in North American media in relation to same-sex benefits, legal protections, and organizational policy and diversity education initiatives, and guides to support transition in the workplace are becoming increasingly visible [10, 12, 13]. Yet, workplace support for gender-variant individuals and those who self-identify on the trans spectrum remains highly politicized, even within LGBTQ communities. In Canada, inclusive policy change related to diversity is part of broader social policy and health professional advocacy for equity; nursing in particular has taken leadership in a Canadian context of the professions [1]. However, the policy context in which discrimination occurs for trans people is still fraught. In Ontario, Canada, findings from the Trans Pulse project [10] indicate that there is little known about trans people's work, although it is well documented that underemployment and barriers to education exist. Many are forced to leave education before completing high school due to experiences of harassment, bullying, and violence, yet there is some evidence that despite this, many are able to achieve some level of higher education [8, 10].

Narratives published in print, or online, offer insight into the dynamics of work and health and the complexities of diverse trans people's lived experiences of gender expression and transitioning in a Canadian context [14–16]. In 2008, the Canadian Professional Association for Transgender Health (CPATH) also emerged to offer resources and professional support for those involved in trans health provision, creating a community for trans-identified providers and allies to be visible in a professional context. However, few trans-identified members of the regulated health professions (e.g., nurses) are visible in the health sector and there is limited research that focuses on their work, and even less on their career trajectories, especially in the professional context (this includes both alternative and regulated health providers) (e.g., [17]).

Yet, gender and its intersections with race, sexuality, and other social categories inform a body of research on professionals and the nature of their work; dominant notions of masculinities and femininities as well as gender conformity have also been noted as relevant to LGBTQ health professionals' career/work dynamics (e.g., [18–20]). As a result, research that fosters an understanding of factors that influence the career, work, and health of diverse trans providers is needed to illustrate the complexities of their lives with the goal of creating supportive policies and practices.

In a nursing context, recent attention to the need to broaden understandings of culturally competent care in order to meet the diverse and holistic needs of patients and clients has pointed to the importance of addressing sexual diversity, and to some extent, transgender health [21–24]. Yet, when this is addressed, most health studies, including nursing research, focus on the context in which trans people are assumed to be patients or clients. By default, the assumption is that all care providers are not trans-identified, but cisgendered (a term used to describe people who are not transgendered [22]) and this has important consequences for the ways in which the health issues of diverse trans people are identified and articulated, and thus the practice strategies that are deemed relevant to supporting them. In fact, a search of the literature using CINAHL, PubMed, Social Science Abstracts, and Psychinfo databases using the terms “transgender” and/or “transsexual” and “work” in titles and abstracts yielded a handful of articles that focus on transgender or transsexual people and their workplace experiences. Typically across the health literature, transgender people and work get taken up in the context of sex work, sexual risk for HIV/AIDS [25] and/or in relation to how providers “work with” diverse clients in ways that often objectify them as “cases.” A recent study suggests that such an approach erases the complexity of trans people's lives [22, 24]. A paucity of workplace-focused health literature exists that offers insight into trans people's workplaces in relation to experiences of transitioning, and/or relevant policy dynamics; in effect, their lives are equated with a patient context rather than a work context which has limitations to understanding health in a social determinants approach.

This paper foregrounds the experiences of health providers who themselves are trans-identified and illustrates the links in their work, careers and health and wellbeing, and the complex ways that gender is implicated in their lives. In particular, this paper focuses on how transmen who are health providers make meaning through their careerlives, that is, meaningful life activities encompassing work and personal endeavors, with implications for their health and wellbeing.

### 1.1. Purpose

This paper explores intersections of gender, health and work or career for transmen health providers in relation to their everyday lived realities. In particular, this paper explores two research questions.

1. How do transmen who are health providers make meaning through their careerlives?
2. What are the implications for their health and wellbeing?

## 2. Materials and Methods

### 2.1. Comparative Life History Methodology

This qualitative study used a comparative life history methodology design. Situating this study in the naturalistic and critical paradigms was appropriate for this exploratory study that examined sensitive dynamics of identity, work and career, and complex aspects of power. This life history approach attends to processes of narration, collaboration, and contextualization so that the researcher has an active role in situating participants' narratives in the larger sociocultural, political, economic, and historical contexts [26]. Critical gender analysis of career histories, within-case and cross-case analysis, can also identify critical incidents and influences, such as identity, that shape diverse professionals' careerlives [18]. In this approach to policy research, the goal is to generate understandings of policy and related processes and practices through the eyes of policy actors themselves in the field [27]. For trans professionals and health providers whose work lives are shaped by policy and whose work entails practices of implementing and creating policy, their lives as trans people are also inscribed by human rights and organizational policies that shape the ways in which trans people are variously included, marginalized, and/or legitimized in their work and larger social worlds [22, 28]. In this study, careers are conceptualized as moving beyond the organizational context to encompass meaningful life activities across the lifespan [29].

### 2.2. Sex/Gender-Based Analysis

Gender-based analysis in this paper, addresses both sex, biological differences between women and men, and gender, which takes into account the social dynamics or gender roles, identities, and norms which shape individuals' holistic health. Historically, sex has referred to biological characteristics that were used to determine discrete binary categories of female or male. However, trans people's attention to and experiences of gender variance and the ways that their biological characteristics are at odds with their sense of being male, female, and/or expressing their gender, blur the discrete boundaries of sex and gender. For transgender people, for example, binary categories of male and female are inadequate to represent the fluid and socially constructed nature of sex and gender. To understand their health requires simultaneous attention to the ways that gender norms influence gender expression and identities [22, 30]. Rather than an exclusive focus on health risks, a critical gender-based approach foregrounds trans people's agency, the ways that they negotiate their social worlds in particular times and places, and material and social dynamics characterizing their careerlives and the ways that gender intersects with work and other social determinants. It also encompasses how both material (e.g., physical embodiment) and social dimensions (e.g., identities, norms) are gendered. Although gender-based analysis has typically been used to examine how gender matters in the lives of cisgendered women and men, Johnson et al. [30] explain that it could be applied to understand transgender/transsexual issues, and more recently it has been taken up in health research on trans people [21, 22]. In this study, we incorporate this gender lens from the outset of the study [30], mindful of the ways that this methodology influences the way the study is conceptualized and questions asked, the data collection, analysis, and sharing of findings. As a type of feminist research, there is also an assumption that the research has political goals with an aim to improve the everyday lives of transgender people.

### 2.3. Procedures

Ethics approval from York University Research Ethics Board was received prior to commencing the study. At that point, a call for advisory group members for the study was sent out, and two trans-identified community members responded and provided feedback on the study materials and procedures. These two community members represented transwomen's and transmen's communities and had insight into health and social service delivery as providers in paid and/or volunteer roles; they opted not to be interviewed, instead showing their support for this study in this advisory capacity. Materials were revised with respect to the language used in the consent forms/recruitment material and the way that the researcher specified how she was situated with respect to the trans communities. The principal researcher, a health professional, conceptualized the study and was involved with recruitment, data collection, analysis and presentation of initial findings. The authors of this article, both of whom been involved with LGBTQ advocacy and research, collaborated on analysis and developed this article. The call out for participants was for individuals who self-identified as “trans health providers” working in the health and social services sector initially in Ontario; and at a later date, recruitment was expanded to include those working in Canada. Thus the study aimed to recruit both health and social service providers who were self-identified as trans (e.g., transgender, transsexual, Two-Spirited, genderqueer) and cisgendered providers who were involved in service delivery for trans communities and thus were key informants. This paper, however, reports on the trans-identified providers and specifically, trans providers who self-identified as transmen. Information about the study was posted through professional and LGBTQ networks in Canada. This included Rainbow Health Ontario and Rainbow Health Network online listserves which have broad reach to gender diverse communities and health providers throughout Canada, as well as the researcher's well-developed professional networks that included a focus on health equity-related research.

The interviews focused on career histories, everyday activities, experiences of health and work, as well as factors that shaped career and workplace decisions and practice dynamics. Interview questions were created for the study based on the literature and the principal researcher's engagement with issues that trans health communities had previously identified as relevant [6]. Interview questions focused on the everyday lived experiences and challenges shaping many trans people's worklives: gender presentation, transitioning, health concerns, discrimination, violence, underemployment, or unemployment, and social support. For example, the researcher asked about a typical day in their life as a trans-identified health provider, pivotal incidents, or key influences that played a part in their health, as well as workplace dynamics, policies, or issues that would contribute to trans providers' health and wellbeing and their vision for strategies that could improve practice environments for trans providers. The advisory group affirmed the focus of these questions in relation to the purpose and scope of the project.

Face-to-face or telephone semi-structured interviews were completed from April 2008 to April 2010 with four participants who self-identified as transmen. Probes were used to encourage participants to provide thick description and elaboration on the context of points raised [31]. These interviews lasted from 1 to 4 hours and were audiotaped and professionally transcribed. Participants were offered a small honorarium. Steps were taken to protect confidentiality and privacy. This was especially relevant given the small pool of “out” trans-identified people in professional positions whose identification could have enormous personal and professional implications, including career risks.

Conventional content analysis [31] was chosen as an inductive approach, a method that is appropriate for examining qualitative data for a study that is exploratory and whose aim is to describe a phenomenon which is not well understood. Analysis of descriptive data emerging from the interviews was guided by the gender-based analytical approach with its focus, for example, on social dynamics, gender norms, and experiences of gender variance. Both researchers independently coded the transcripts for themes and subthemes that were guided by an understanding of gender dynamics (e.g., the notion that both material and social factors are relevant to worklives). However, the researchers were also attuned to unexpected themes that emerged, such as some participants' experiences of congruence between their work interest and their social identities as members of trans communities. Transcripts were read as a whole for immersion in the data and then line-by-line coding was carried out in which key phrases were identified as initial themes and notations made with researchers' reflections on emerging codes, “allowing the categories and names for categories to flow from the data” ([31], page 1279). Where possible, verbatim phrases were used as codes. For example, we identified one code as “gender identity as career" based on one participant saying, “*being able to transcend the limits of um physicality*…* my gender identity is my career in a sense*…* or vice versa you know*…*. I see both of these unfolding more in the future.*” This code later became part of the theme “Fitting In". Under the theme of *Critical Influences*, we identified a subcategory: “*feminist work that influenced me most in terms of identifying as queer and coming out as queer.*” Researchers identified themes, codes, and subcategories which were later clustered in a coding scheme. The two researchers shared these initial themes, which were then validated using consensus process and were informed by the existing literature. When there was disagreement on emerging themes, discussion ensued with return to the transcripts until the researchers reached consensus. Thus the way gender-based analysis was used in this study considers the ways that gender normativity provides a reference point for understanding the experience of people who do not fit into the binary categories of male and female. As well, it affirms the unique experiences and identities of diverse trans people “who share the common experience of knowing themselves to be a gender that is not congruent with their birth sex” ([21], page 59).

### 2.4. Sample

In this paper, analysis focuses on the career histories of four health providers who self-identified as transmen. These participants, who ranged in age from their 20s to their 50s, had varied gender histories and experiences from those who had transitioned recently, to those who had done so over 10 years previously. All currently live and work in urban centers in Canada. The narratives represented experiences of students in professional and alternative health programs and providers with decades of work experience, some also with international experience. Professional work and/or education over their lifetimes included social work, medicine, nursing, midwifery, naturopathy, massage therapy, as well as other care work such as teaching. Because of the extremely small pool of openly trans-identified health providers and specifically those with professional credentials in a Canadian context, narrative excerpts and findings are reported in this paper in ways that will not identify participants; information such as age, profession, and place of work are omitted. TM identifies the participant as a transman, and a page number from the transcript is cited.

## 3. Results and Discussion

In this paper, we report on themes that represent how participants articulated gender identities in the context of work, their careerlives and larger lives, so that the focus is on meaning-making through gender/transition and “doing” gender in their work and other meaningful life activities. Themes relate to their engagement with their trans identities and careerlives with implications for fostering or challenging their health and wellbeing.

### 3.1. Gender(ed) Identities

All participants described themselves in ways that identified themselves as “transmen.” Over their lifetimes however, they reported quite different identities, which, in part, shaped and were shaped by their particular engagement with gender identities, transition processes, and other ways in which gender factored into their social worlds. For instance, deeply entrenched homophobia, heterosexism, and biphobia contribute to gender norms and invisibility, lack of awareness, and transphobia as factors that hinder diverse trans people's ability to locate respectful, responsive, and meaningful health care in their lives [7, 22]. These narratives point to the ways that such dynamics can also influence their narratives of becoming aware of a masculine trans identity as relevant to their wellbeing. All four participants shared that their engagement with a trans identity occurred as adults, with three having also incorporated a same-sex identity for most, or part of their teenaged years. For instance, while coming out and exploring his bisexual and queer identities in his late teens, one of the participants noted that he

*“met a very different gendered world…. It was all really androgyny…. At first I didn't really want to do it, but it was very helpful in the end, because I didn't feel that pressure to conform to that gender world anymore, so I could come out and go out to something more myself in a different way.” (TM, page 18).*

Another described the initial “*trigger… not from becoming attracted to someone, which I think is not the usual way… just the access to those kinds of books, or the encouragement to read books that were exploring gender identities and sexuality*” (TM, page 9). For yet another participant, this awareness emerged in more gradual and complex ways:

*“Who am I really? And what choices can I make to be more at ease with myself? I don't want to be a woman like this anymore, because it's not working for me… people are rejecting me as I am and I'm being rejected by my partner and I can't be a mother in the way I want to because of my career. And I started to think, well maybe I was just made atypically, you know.” (TM, page 26).*

In each case, taking on a trans identity was incredibly affirming for these participants on a personal level, even as they encountered unpredictable challenges for disclosure and gender expression, which prompted both feelings of elation when things were working well and also at times, deeply wrenching emotional upheaval. Moreover, their narratives illustrated how their education into a profession and/or workplace context was linked to these dynamics.

### 3.2. Material and Social Factors Shaping a Career as a Health Care Provider

Several participants spoke of what drew them to health and care-related professions. As one noted, the journey of becoming queer-identified “led me into the health sector was… wanting to take care of my own health” (TM, page 4), while another suggested that trans people “definitely… have… a better chance of making it in health care or social services.” He suggested that “it can be helpful for trans clients…. Having a trans- [identified…] worker… would make sense for them, but you know that is not… common” (TM, page 14). In these ways, transmen in this study proposed that their careerlives and career choices represented a holistic, or spiritual component rooted in their gendered identities and their experiences of experiencing marginalization in social institutions.

Financial factors surfaced for some participants who acquired debts from education or weighed transition-related costs on top of living and/or education expenses. Working in contract positions or in alternative health contexts, which were not publicly funded, prompted some to take on heavy workloads and juggle several jobs—both in health or research and those that were not health-related. Such pressures exacerbated participants' everyday stresses.

### 3.3. Transitioning on the Job/School: Materiality of (Gender) Change

At times, the connection between work and identity was complicated by the material consequences of transitioning. Pressures to conform to rigid gendered expectations for roles and appearance carried over to their presentation in professional contexts and the unease that they experienced. As one participant shared, part of his transition included a gradual change in dress and appearance while working and this visibility in relation to his gender expression was an important step towards enhancing his psychological wellbeing:

*“So, I kind of on the one hand rejected traditional… womanhood I suppose… and I started to dress in a way that made me feel comfortable for the first time. I used to have long, long hair and I cut all my hair. I cut my hair and I started to feel much better” (TM, page 27).*

Another participant pointed out some of the challenges of hormonally transitioning while working, indicating that he often avoided interactions with his peers because his voice was not yet low enough for him to feel confident that he wouldn't be singled out, noting, “I didn't want them to notice me, basically, and I definitely didn't want them to think poorly of me” (TM, page 40). Another related that while commuting to work during the transition period he experienced negative incidents riding the bus: “like… the bus driver being rude, you know, or interacting with someone who's being transphobic…. Sometimes I'd come in… demoralized before the day even started.” (TM, page 12). Further, he added that while, “wearing pants to work was a blessing” when transitioning from female to male generally, but having to ride a bike to work to save money presented issues when binding his breasts. “ I couldn't breathe… with all the hills…. I couldn't bind and ride my bicycle so…. I had to go into work and change…” (TM, page 12).

Several participants also shared stories of the struggle to get registered as a working professional or to apply to a professional program. In each case, this professional context was a turning point for the provider. Working and transitioning, whether as a credentialed professional or professional-in-training, meant that these transmen had to worry about how to change their documentation and professional registration, in addition to dealing with the multiple decisions related to, and demands of, disclosing (or not) and living and working as a transman in various community and work contexts. As a result of this, application to professional schools and professional bodies was fraught. As one participant shared, “The struggle to get registered… was like a warning of how hard it was going to be” (TM, page 25). Another participant justified the timing of changing his name at the beginning of his professional career having suddenly realized that, “you start meeting [colleagues] you'll be working with… people who… you'll want the support of for the rest of your career…. I should be ready to go as the person that I want to be for the rest of my life*”* (TM, page 15).

In terms of the work setting, another explained the complicated nature of having to determine how and when to disclose professional education and experiences obtained pretransition:

*“Before, I was, you know, passing when I was first transitioning…. What I struggled with was when to come out. Because if you put it on your cover letter you might not get called, but if you don't address it fairly on when you show up for the interview than they sort of look at you and they call your references…. And especially in social… health care… your reputation is, like, being able to verify your character is a very important part of people being able to hire you, right?” (TM, page 8).*

In response to the question, “Why didn't you use your legal name?” by an administrator, one participant reported that he disclosed being trans, saying, “Well I've been like transitioning, not physically, but I'm in the process of questioning my gender identity and I feel more comfortable with [chosen name]” (TM, page 28). Although such processes were eventually resolved for this participant, disclosure of trans identity had significant negative impacts for other participants. Two participants reported that disclosure engendered larger questions about their credibility, mental health, maturity, and fit for the profession itself. One of the participants described that he

*“had a meeting with… support [staff…who] asked me if this was the right time for me to be in [professional] school… until I had sorted out my gender and like until I could pass as male, did I really think that this was the right time for me to be here?” (TM, page 53).*

At times, the struggle to disclose or provide professional documentation was avoided by changing their legal name only and deferring gender transition until later. Participating transmen also reported having to move jobs and/or change careers to be able to work in their chosen genders. One participant further illustrated that even in a professional school environment that offered the potential of strong group support, disclosure and transition were not straightforward:

*“I was… coming out in my personal life in my second to last semester and so then in my last semester I was doing my practicum and I wasn't quite confident to share that with the whole school…. It seems like that was too much outing to deal with at once and I knew that they would have to process it because we were social workers.” (TM, page 10).*

### 3.4. Education and Work Environments

With few exceptions, trans-identified providers are invisible in health care. Those affiliated with mainstream education programs or work settings such as hospitals were thus likely to be the sole trans-identified provider in their workspaces. Professional isolation in particular, emerged in several career histories, with experiences of physical and mental distress as a result. This was a pervasive dynamic for at least one participant in several workplaces, exacerbated by the nature of the precarious work and related working conditions, and by the need to move to different job locations in order to find work and support the family: “It was just an incredible experience… just feeling utterly alone… through the whole year. Actually, professionally, I didn't really have any supports” (TM, page 21).

The experience of transitioning at work or in educational contexts meant that at times trans people's health suffered with the potential for adverse outcomes. One spoke of a long period of recovery after several spells of serious illness which made it impossible for him to work: With “no sick benefits [or] money… to tide me over…. I was homeless for a period” (TM, page 28). Another shared,

*“It was very hard…. I was coming up against the health care system and not being able to… get started with transition and then… being all stressed out at work… and obviously in my personal life as well going with lots of stuff at the beginning of transition…. I was underweight and coughing and didn't sleep well…. It was definitely impacting my mental health… my physical health too…. I was not thriving” (TM, page 7).*

Participants spoke highly of allies they encountered in professional and educational contexts, who often, but not always, were members of LGBTQ communities. This played out in various ways, where individual mentors and champions advocated on their behalf, provided important emotional support, challenged systems to become more responsive to their needs and provided role modeling in clinical placements where they could engage with trans health. Participants in particular identified that being trans and working in health care meant that education and “coming out” were constant worries or struggles, and that employers who supported them through medical transition were highly valued. “I was very lucky… working in a health care agency…. They were very understanding that I needed to go to the doctor constantly” (TM, page 3).

At times, some of the participants could also tap into LGBTQ professional networks in their work and educational settings, which relived some of the isolation and marginalization that they felt:

*“It really kept it all together for me in… school because it can be overwhelming and it's isolating to feel like you're the only person who is like you. You know, there's no other trans people that I've encountered working…. So, going to [a LGBTQ-positive agency] really just re-energized me for the rest of the year.” (TM, page 46).*

Yet, their career histories showed that such support and resources were not always consistent, even within the same organization, and this created significant anxiety either throughout their experience, or at particular points. This prompted one participant to take action using a formal complaint process, despite having a supportive manager, remarking that his colleagues were “making inappropriate remarks towards me…. I'm getting upset and that's not fair” (TM, page 5). Transitioning at school, or living as a transman outside of work, also posed challenges in intensive programs where students were immersed in environments with professional colleagues and fellow students. Physical spaces also contributed to affirming or nonaffirming settings for individuals. As one participant shared, “There's just no space for me, you know, and the locker rooms… even us students… some of them are both genders but some of them are not” (TM, page 28).

Working in professions which were historically known for being female-dominated, or which had strong male roots, especially in institutional settings, prompted concerns more often than working in alternative health or small agencies that focused on the care of highly marginalized populations. One participant who worked in such a context noted that, “I was afraid I would have someone who would refuse to call me “he” or like… be ignorant or discriminatory, and, I just lucked out. These are the things that make people believe in, like, a higher power” (TM, page 45). Another participant, who had experienced workplace bullying and scapegoating, remarked that, “the more structured and hierarchical an organization is, the more you need to be just like a little cookie cutout… so everybody is comfortable…. It's one less thing to worry about in the high stress situations, I guess…” (TM, page 7).

### 3.5. “Fitting In”

Lack of fit within the profession surfaced as a theme for participants both before gender transition began, and afterward. At times “lack of fit” was also used by employers to discriminate against participants after they began to transition, and this influenced job opportunities and job security. As one participant explained:

*“‘the glass ceiling'…. There's a point beyond which you can't reach and it's kind of invisible and you just kind of grind to a halt and there's no real explanation… you're nice, dependable… but there's something that just doesn't quite gel.” (TM, page 6).*

He recalled being stunned by employers' feedback. “They said, ‘We just don't feel you're a good fit.' And I asked them to elaborate but they absolutely couldn't” (TM, page 23).

All of the narratives revealed instances of explicit work-related discrimination or challenges of “fitting in” that were insidious. One participant, who perceived discomfort from peers when he began a new job, questioned how much education his current coworkers had about working with trans people on a team: “I'm a tranny but no one seems to process that…. I don't know if they are totally blasé or nervous” (TM, page 8). Another participant received a clear message from several higher-ups that gender transition in the professional training context was a weakness. Their attitudes conveyed that “people need to give you a hard time about it until you're tough enough, you know, you're thick-skinned enough that it won't bother you” (TM, page 34).

On the other hand, fit was also associated with a positive change after transition consistent with processes that engendered participants' resilience and growth. For some, “fit” meant that how they did their job changed after transition because they identified a new niche for their practice, or a different way of doing gender that added a new element or a new community. One participant explained that he felt that he “does masculinity” differently than other men and that this is advantageous in his professional work with men who often have complex health concerns and who seek him out to discuss tough issues that they would not discuss with straight male or female providers. Another spoke of being “wowed” by the possibility of being able to weave together his trans and professional identities in ways that are valued in a health care context such as participating openly in LGBTQ-positive education, although he recognized that there is still much to be done before this is possible in all facets of his work as a provider: “So that was the beginning of… a new phase…. I'm beginning to integrate my identity with my health care provision” (TM, page 33).

For another participant, career choices involving alternative health care and health services research paralleled his emergent identity that for him were congruent, because both challenged the prevailing biomedical and binary discourses in health in ways that not only fostered the creation of trans-positive workplaces and professional practice, but also had profoundly positive consequences on a personal level. His narrative shows links between meaning-making in a career context and the process of coming to identify as trans:

*“My gender identity is my career in a sense… or vice versa you know…. I see both of these unfolding more in the future and yeah…. I've, I feel that it is also significant that I have come to both of them at a similar time, like I feel like my life has really changed course as I've… um shifted as how I identify as both my sexual orientation and my gender identity….” (TM, pages 7-8)*

Certainly, the opportunity to find supportive communities both within and beyond their workplaces where participants could validate their holistic lives and identities was enormously valuable for wellbeing. This was represented by one participant who shared, “I'm not going to go stealth. I don't want to erase my past as a female and I think that being trans gives me a unique position in the world and…. I want that to be known” (TM, page 75). Another spoke of connecting to “queer community [through] freelance writing and… through the gender expression performance” (TM, page 9). Yet for another, his decision to disclose as a transman, which was sparked by professional concerns, prompted unexpected ripples of family support. “Well I'm thrilled… everyone is just okay with it” (TM, page 14). Finding community or re-reconnecting unexpectedly with colleagues or family surfaced as significant effects of the processes of expressing trans identities for all participants in some context, although support varied considerably among the narratives.

## 4. Discussion and Implications

This life history study, with its attention to analysis within and across narratives, points to critical events and influences that shape trans health providers' engagement with their identities as transmen and as health providers. Gender and sexuality are determinants of health that are relevant to diverse trans people, whose everyday lives reflect a continuum of femininities and masculinities in a context of cisnormativity. Cisnormativity, which is embedded in all social institutions and our everyday worlds [32], refers to the assumption that all people are cisgendered [22]. In these narratives, gender and work emerged as the most salient social determinants in trans people's career and worklives. 

The findings of this study show that trans people have unique, and gender-related, critical events and influences that shape continuities and discontinuities in their careerlives. Examples of continuity are reflected in their decision to remain involved in health provision despite experiencing immense challenges brought on by experiences of discrimination, harassment, and ill health. Despite this, the narratives of the participants also reveal that at times their career trajectories were curtailed or limited by these challenges, and for some eventually necessitated an unanticipated shift in the scope of work or a change in health profession.

Both social and material factors figure in their gender articulations and in their careerlives. In fact, these narratives show how personal and professional identities, in conjunction with practices of gender expression, are intimately linked to professional socialization processes. As a result, this study adds to the necessity of expanding organizational and educational environments by bringing forth trans providers' narratives and experiences. As one participant indicated, it is particularly crucial to “bring […] forth trans voices that have this reality made known to a wider audience” (TM, page 3) with a focus on gender fluidity and as opposed to sexuality, especially in a context of health care where transmen are often invisible.

The focus on ongoing transitions without an endpoint that was demonstrated in this study challenges the more dominant before/after transition models of trans people's lives with stages (e.g., [33]) and this is consistent with what we have foregrounded in this paper as a more fluid approach to gender and career/work transitions more broadly. The narratives revealed that both the professional and personal lives of trans providers reflect continuities and discontinuities that have particular consequences for their career and worklives. Although continuities between the personal and professional may be relevant to a variety of individuals, findings of this study suggest that these continuities and discontinuities also have health-related effects that are both health-promoting and health-depleting—in effect—deleterious.

In fact, the intersection of work, gender, and health are clearly depicted through these narratives, in terms of employment, working conditions, and professional culture, which all have implications for nursing. For instance, although it is crucially important for nurses to open spaces and contribute to the development of workplace policies that will foster transition processes for providers, as these findings suggest, ongoing workplace support of diverse colleagues encompasses a range of practices that move beyond the transition process. These include an awareness of the everyday challenges of living and working in one's chosen gender, the significance of one's chosen profession as it connects to one's trans identity, and the importance of interpersonal, organizational, and professional supports to enhance broader community and professional environments for trans people across their lifespan. Thus these findings bring to bear a complex understanding and novel way of exploring health for trans people as workers and in particular as health care workers.

While we appreciate that there is an assumption that LGBTQ health care providers experience similar workplace concerns in general, such as stigma, management of identity and social exclusion of minorities that factor into career trajectories and a range of workplace-related issues such as health, social support, and policy, we maintain that such literature is limited in its relation to our study's focus on the career/work context. In fact, some LGBTQ-focused literature on the workplace appears to address trans issues and focus on trans people; however in effect it actually elaborates on sexual orientation rather than gender identity. For example, Bell et al. [34], while offering important insights into workplace dynamics for sexually diverse groups, do not address the unique needs of trans people in comparison to LGB people, despite a title that espouses a focus on LGBTQ workplace concerns. In fact, Brewster et al. [35] call for the development of transgender-specific versions of vocational measures to study trans workplace experiences given the ways that LGBQ-focused measures are insufficient to capture the unique issues of trans people. As they note, “many of the issues discussed in LGB vocational scholarship may be pertinent to transgender people, but the unique manifestations of… experiences of workplace harassment and discrimination, workplace climate, and identity management… are shaped specifically by gender identity and presentation” ([35], page 61).

Thus, a key contribution of our paper is the juxtaposition of career, work, and health for trans-identified people who are health and social service providers, and in particular the gender-based analysis with its focus on transgender people is unique. This paper complements the main foci of health literature on trans people which assumes that they are clients, and the workplace literature which mainly addresses gender transition and related policies—important as they are to trans people's worklives (e.g., [10, 12, 17, 36]). The findings also extend the dearth of trans-focused literature in the counselling and vocational literature (e.g., [11, 33, 35–39]).

This study contributes to a small, but increasing, body of nursing and health professional studies on gender diversity in clinical care, cultural competence, and gender nonconformity in the professions (e.g., [19, 20, 23, 25, 28, 40–44]). The findings build on research on transmen and trans people's work experiences which address transition policies and practices, identities, disclosure, discrimination, and employment counseling (e.g., [17, 37, 45, 46]), and on LGBTQ-affirmative counselling and LGBTQ-positive climates in higher education (e.g., [39, 47]). The one nursing study that examines workplace experiences of LGBT-identified nurses in an American context [17] offers important survey data on three respondents who are trans-identified. Short narrative insights from these participants and same-sex-identified nurses as key informants indicate that trans nurses encounter challenging workplace dynamics with some positive support noted. Our contribution, a gender-based analysis of qualitative data, in-depth interviews with transmen whose lifetime careers represent a variety of health care professions, including nursing, offers new and unique insights. Given the significant gap—a silence—in the literature in relation to trans people who are health care providers and the gap in nursing publications in relation to trans issues in general, this study provides much needed information.

Without a doubt, making visible the career histories of these, often invisible, trans providers in ways that show resilience and complexity, provides not just stories of role modeling for other trans people and other marginalized people in the professions, but also acts as a counterpoint to the often grim evidence base that comprises trans health. Certainly, professional education and activism that aim to alter structural dynamics are needed to foster the creation of equitable spaces for diverse trans people to thrive as health providers (e.g., [7, 17, 47]).

These findings address the invisibility of trans people as health providers rather than as clients in ways that are strength-based and move beyond a focus on marginalization and mental health concerns. This qualitative research study provides thick description and illustrates the specific and multiple contexts of trans people's lives that show how challenging employment settings, poverty, ill health, and marginalization may be part of trans health professionals' life experiences. Yet, these are also positive narratives of people who are successful in their lives and careers and who recognize their privilege as trans people who can work and thrive in particular professional contexts. Certainly this small exploratory study of a selected group of trans providers who have had access to professional training and/or acquired professional credentials and who are working in the field reflects trans people with high social privilege and does not aim to represent all providers' or transmen's experiences, but instead aims for understanding. More research is needed to examine a range of trans people's careerlives that accounts for a diversity of identities, practice settings, and ethnoracial differences, for instance.

The findings show how work and career are mutually informing, and how work is meaning-making or community-finding—all of the participants talk about finding or establishing a community at some point. These narratives also point to the negotiation, compromise, and resistance that figure into their everyday decision-making in the education and workplace environments where they are doing this gender work. As they work within structures that are shaped by gender normativity and which prescribe “fit” within professional practice, they also often push the structures to change through their visibility when they are able. These are not client or patient narratives of transition or of being trans, but rather these are insider narratives of the health profession that expand the spaces for talking about gender and transgender in health care contexts [48, 49].

Meaning-making through work and career, and finding community through gendered practices, resilience, and lack of linear trajectory are about process rather than an endpoint. Such career experiences in a professional environment are characterized by both continuities and discontinuities, as noted earlier. The findings extend what is typically discussed about the physical and emotional upheaval that transition can bring. By foregrounding discontinuity we see multiple critical incidents and influences in trans people's personal and professional lives that complicate what is often represented as a linear trajectory of professional work and career decision-making in general, and for trans people more specifically [33, 46]. As transmen negotiate their work and identities, making meaning through their career paths, they are un/doing gender and doing transgender [46]. This work builds on the work of West and Zimmermen [50] on doing gender, and Connell's [46] extension of that in relation to the ways in which transgender people do “transgender work” by foregrounding the lived experiences of providers who are transmen who experience policy “on the ground” both as “clients” of health systems and as policy actors within health systems.

The focus on spiritual values [51, 52] in a professional context, which has often been in opposition to a medical model, is consistent with a holistic and hopeful framing of transgender that informs these participants' expression and articulation of their trans identities and practices as providers. This spirituality component in its focus on meaning-making is a unique contribution to the literature on gendered identities for trans people. Relating both to themes of continuity and “fitting in,” for some of the participants, gender identity and transitioning have catalyzed an expanded meaningful and positive connection to their professional identity/role. In spite of discontinuities that they experience in relation to identities, and at times experiences of rejection in work and/or professional environments, there is a congruency in the ways they create career paths in relation to their practices as health providers [52]. They are working inside and outside the work context in political ways to challenge gender norms in the ways that they participate in professional, queer and/or trans community politics, connections and professional education; Connell [46] would suggest that this is doing transgender work. There are thus implications for nursing research, education, and practice in terms of understanding how trans providers “do transgender work” and supporting them in that process.

Certainly, research and educational strategies which make visible the lives of trans people as care providers in a nursing, and larger provider contexts, are important steps; trans-inclusive education and training for cultural competence to enhance individual nurses' awareness, as well as organizational support, are warranted in order to create positive working environments that support providers who are trans-identified. Such dynamics are intimately linked to nurses' capacity to practice in inclusive ways as advocates for sexual and gender minority clients and communities. As indicated in life history research with other diverse nurses, these work and practice links have health implications that point to fostering providers' wellbeing as well as their capacity to provide responsive care [19, 42]. In order to create trans-positive spaces, findings of this paper support the development of a working paper on the process of formally integrating trans providers into the professional context. This paper thus takes up the call by Eliason et al. [53] for emancipatory nursing research which examines more fully the complexity of diverse LGBTQ people's lives.
